# Relationship Between *Histomonas meleagridis* Infection and Cecal Intestinal Microbiota of Chickens

**DOI:** 10.3390/vetsci13020118

**Published:** 2026-01-27

**Authors:** Qiaoguang Chen, Yaxin Liu, Wendi Zhu, HsuPan Aye, Ruting Li, Zhaofeng Hou, Dandan Liu, Yuelan Yin, Jianping Tao, Jinjun Xu

**Affiliations:** 1 College of Veterinary Medicine, Yangzhou University, Yangzhou 225009, China; 008919@yzu.edu.cn (Q.C.); mx120231010@yzu.edu.cn (Y.L.); mz120221616@yzu.edu.cn (W.Z.); 712001@163.com (H.A.); mz120241764@yzu.edu.cn (R.L.); zfhou@yzu.edu.cn (Z.H.); ddliu@yzu.edu.cn (D.L.); yzjptao@126.com (J.T.); 2 Jiangsu Co-Innovation Center for Prevention and Control of Important Animal Infectious Diseases and Zoonoses, Yangzhou University, Yangzhou 225009, China; yylan@yzu.edu.cn; 3 Ministry of Education Key Lab for Avian Preventive Medicine, Yangzhou University, Yangzhou 225009, China; 4 College of Bioscience and Biotechnology, Yangzhou University, Yangzhou 225009, China

**Keywords:** *Histomonas meleagridis*, gut microbiome, probiotic, microbial diversity, 16S rRNA gene

## Abstract

Histomonosis, caused by *Histomonas meleagridis*, leads to economic losses in the poultry and livestock industry. In recent years, studies on the role of intestinal microbiota in host physiological health have attracted growing attention. Understanding the changes in gut bacterial communities of chickens is crucial for improving poultry and livestock production. This study investigated the impact of *Histomonas meleagridis* infection on the growth performance, overall health, and cecal microbiota composition of chickens. Body weight changes and pathological alterations were assessed at different time points post-infection through animal experiments, with 7 days post-infection defined as the early stage and 14 days as the peak stage of infection. Cecal content samples were collected from the 7-day control group (G1), 7-day infected group (G2), 14-day control group (G3), and 14-day infected group (G4) for 16S rRNA sequencing analysis. The microbial diversity analysis revealed that *H. meleagridis* infection altered the number of microbial species in the cecal microbiota of chickens. The alpha diversity index was significantly reduced (*p* < 0.05), and principal coordinate analysis (PCoA) revealed significant structural differences between infected and control groups (*p* = 0.001). Taxonomic composition analysis showed that beneficial gut bacteria, such as Firmicutes and *Lactobacillus* spp., decreased in abundance, whereas Bacteroidota, Proteobacteria, *Escherichia* spp., and *Fusobacterium mortiferum* were enriched in the infected group. LEfSe analysis indicated that G1 was enriched with *Oscillospiraceae* and *Blautia*; G2 with *Christensenellaceae*; G3 with *Verrucomicrobia* and *Lactobacillus aviarius*; and G4 with Proteobacteria and Fusobacteria. In conclusion, *H. meleagridis* infection markedly altered the cecal microbiota composition by shifting the relative abundances of beneficial and pathogenic bacteria, resulting in reduced microbial diversity.

## Simple Summary

This study evaluated the effects of *Histomonas meleagridis* infection on growth performance, lesion indicators, and the composition of the cecal microbial community in chickens. The results showed that fourteen days post-infection represented the peak stage of disease development. Compared with the control group, infected chickens exhibited a significant reduction in body weight, along with severe lesions in the liver and cecum. At the same time, the abundance of beneficial bacteria in the cecum, such as Verrucomicrobia and *Lactobacillus aviarius*, decreased, whereas the abundance of harmful bacteria, including Proteobacteria and Fusobacteria, increased. These findings contribute to a better understanding of microbial changes in the chicken cecum following infection with *H. meleagridis*.

## 1. Introduction

*Histomonas meleagridis* is a protozoan parasite responsible for histomonosis, commonly known as “blackhead disease” or infectious enterohepatitis [1,2]. Characteristic lesions occur in the liver and cecum, typically presenting as caseous cores in the cecum and volcanic crater-like focal necrosis on the liver surface [3]. The disease poses a serious threat to turkeys, with mortality rates reaching up to 100%. In chickens, while the mortality rate is comparatively lower, histomonosis significantly impairs production performance, resulting in decreased egg production, reduced body weight, and an elevated mortality rate that can persist for several weeks [4,5]. In recent years, concerns regarding food safety and public health have led to the progressive ban of nitroimidazole compounds, nitrofuran compounds, and other effective veterinary drugs in the United States, the European Union, and China [6,7,8,9,10]. The live attenuated vaccine obtained through serial in vitro passaging has demonstrated effective immunoprotection but has not yet been successfully commercialized [11,12,13]. This situation has created significant challenges in the prevention and control of histomonosis, necessitating urgent exploration of novel strategies. Previous in vitro studies have shown that *Escherichia coli* in the gut influences the energy absorption and metabolism of *H. meleagridis* [14], while in vivo research has revealed that the presence of *H. meleagridis* increases the relative abundance of *E. coli*, thereby exacerbating pathological changes [15,16]. Thus, elucidating the interactions between *H. meleagridis* and intestinal microbiota populations may provide critical insights for developing novel alternative therapies.

The intestinal microbiota refers to the microbial community inhabiting the animal gut, including trillions of bacteria, fungi, viruses, archaea, and other microorganisms [17]. The chicken intestine harbors a highly diverse microbiota that interacts with the host to establish a stable and coordinated intestinal ecosystem [18]. Complex interactions between intestinal microorganisms and the host influence physiological balance, health, immunity, and production performance [19,20,21]. Recent studies have demonstrated that parasitic infections can induce gut microbiota dysbiosis, leading to intestinal barrier dysfunction and exacerbation of disease progression. Infection with *Trichuris ovis* significantly altered the diversity of the cecal microbiota in goats, increasing the abundance of opportunistic pathogens such as Proteobacteria and Bacteroides while reducing beneficial bacteria [22]. *Entamoeba histolytica* infection induced notable shifts in the gut microbiota of amoebiasis patients, characterized by an increased abundance of *Prevotella copri*, which was associated with inflammatory diarrhea [23]. Similarly, in broilers infected with *Eimeria acervulina*, the alpha diversity of the luminal and mucosal microbiota in the duodenum and jejunum was altered, manifested by a reduced abundance of short-chain fatty acid-producing bacteria and an increased abundance of opportunistic pathogens [24]. Infection with *Eimeria tenella* modified the composition and structure of the cecal microbiota in broilers, leading to a decrease in beneficial commensals such as *Lactobacillus* spp. and *Faecalibacterium prausnitzii*, while increasing the relative abundance of *Enterococcus* spp., *Streptococcus* spp., *Escherichia coli*, and *Campylobacter jejuni* [25,26,27]. However, current research on *H. meleagridis* infection and changes in the chicken gut microbiota has primarily focused on the phylum and genus levels, with no reports at the specific species level. Based on this background, the present study investigated changes in body weight, liver, and cecal lesions in chickens following *H. meleagridis* infection, as well as alterations in the cecal microbiota composition at the phylum, genus, and species levels during both the early and peak stages of infection. This approach aims to identify bacterial taxa associated with histomonosis and provide a basis for developing alternative strategies to mitigate the impact of the disease on the poultry industry.

## 2. Materials and Methods

### 2.1. Ethics Statement

All animal procedures were performed in accordance with the guidelines and regulations of the Animal Experiment Ethics Committee of Yangzhou University, under license no. SYXK (SU) 2021-0027 for chickens. All chickens were housed and handled in accordance with the principles of humane care and use of laboratory animals.

### 2.2. Animals

A total of 30 one-day-old white leghorn chickens were purchased from Jiangsu Boehringer Ingelheim Vitong Biotechnology Co., Ltd. (Nantong, China). The chickens were housed in an *H. meleagridis*-free environment, and their feed was supplemented with multivitamins and trace elements.

### 2.3. Parasite

The *H. meleagridis* strain JSYZ-D, isolated from the liver of an infected chicken in Yangzhou, is preserved in liquid nitrogen at the Parasite Laboratory of the School of Veterinary Medicine, Yangzhou University. After resuscitation, it was inoculated into a mixed medium consisting of 9 mL Medium 199 (Gibco, Grand Island, NY, USA) and 1 mL of 10% inactivated horse serum (Gibco), supplemented with 11 mg of sterilized rice starch (Sigma-Aldrich, Shanghai, China), and incubated at 40 °C in an anaerobic incubator [28].

### 2.4. Experimental Design

At 14 days of age, all chickens were weighed and randomly divided into two groups—the *H. meleagridis*-infected group and the control group (after adjustment to ensure similar average body weights across groups). Each group consisted of 15 chickens, housed in separate cages. On the same day (14 days of age), each chicken in the experimental group was artificially infected via the cloacal route with 2.0 × 10^5^ *H. meleagridis*. Prior to artificial infection, the number of *H. meleagridis* cells/mL was calculated by hemocytometer and trypan blue (Sigma-Aldrich, Shanghai, China) staining to ensure the viability of the parasites at the time of infection. Artificial infection was performed according to the method described by Beer et al. [11].

### 2.5. Sample Collection and Intestinal Lesion Score

On 7, 14, and 21 days post-infection, five chickens were randomly selected from both the infected group and the control group at each experimental time point. Thus, all chickens were divided into six groups according to the dissection time, with five chickens in each group. At the three experimental time points, the chickens in the infected and control groups were weighed and then euthanized via cervical dislocation, followed by lesion scoring of the cecum and liver. At each experimental time point, cecal contents were collected from each euthanized chicken, and these content samples were stored at −80 °C. Cecal content samples from the infected group and their corresponding control group chickens with severe lesions and high lesion scores were selected for microbial sequencing.

### 2.6. Lesion Scoring Rules

Cecal scoring criteria [29,30] were as follows: the longitudinal fold of the cecal wall was well-characterized and lacked macroscopical lesions, and the cecal contents were thick with dark feces and no caseous exudate, score 0; cecal wall thickening or presence of scattered petechiae, or both, score 1; moderate thickening of the cecal wall with caseous exudate or contents forming a caseous core, color change of cecal contents or absence of contents and bleeding spots in the cecum, score 2; the cecal wall was thickened, with a prominent caseous core of cecal contents, the cecum had no contents, or the cecal wall appeared petechiae, score 3; the wall of the cecum was significantly thickened, and the cecal mucosal layer appeared fibrotic necrotic and ulcerated, with a caseous core or no contents in the cecum, the presence of a hemorrhagic blind end, or cecal rupture leading to peritonitis, score 4. Liver scoring criteria were as follows [29,30]: no macroscopic round necrotic lesion, score 0; presence of 1–5 small, round necrotic foci (<5 mm in diameter), score 1; many small, round necrotic foci (≥5), or large necrotic foci (≥5 mm in diameter), score 2; many macroscopic small and large necrotic foci, score 3; presented with complex lesions and numerous mixed lesions, score 4. All lesions were scored without knowing the grouping.

### 2.7. DNA Extraction, Library Construction, and Sequencing

Microbial DNA was extracted from cecum contents using the E.Z.N.A. soil DNA Kit (Omega Bio-tek, Norcross, GA, USA) according to the manufacturer’s protocol. The quality and concentration of DNA extract were determined by 1.0% agarose gel electrophoresis and a NanoDrop2000 spectrophotometer (Thermo Scientific, Waltham, MA, USA) and kept at −80 °C prior to further use. The full-length bacterial 16S rRNA gene was amplified with primer pairs 27F (5′-AGRGTTYGATYMTGGCTCAG-3′) and 1492R (5′-RGYTACCTTGTTACGACTT-3′) using a T100 Thermal Cycler PCR thermocycler (BIO-RAD, Hercules, CA, USA). The PCR reaction mixture included 4 μL 5 × Fast Pfu buffer, 2 μL 2.5 mM dNTPs, 0.8 μL of each primer (5 μM), 0.4 μL Fast Pfu polymerase, 10 ng of template DNA, and ddH_2_O to reach a final volume of 20 µL. PCR amplification cycling conditions were as follows: initial denaturation at 95 °C for 3 min, followed by 29 cycles of denaturing at 95 °C for 30 s, annealing at 60 °C for 30 s and extension at 72 °C for 45 s, and single extension at 72 °C for 10 min, ending at 4 °C. The PCR product was extracted from 2% agarose gel and purified using the PCR Clean-Up Kit (YuHua, Shanghai, China) according to manufacturer’s instructions and quantified using Qubit 4.0 (Thermo Scientific, Waltham, MA, USA). Purified amplicons were pooled in equimolar amounts and paired-end sequenced on an Illumina Nextseq2000 platform (Illumina, San Diego, CA, USA) according to the standard protocols by Majorbio Bio-Pharm Technology Co., Ltd. (Shanghai, China).

### 2.8. Bioinformatic Analysis

Raw FASTQ files were de-multiplexed using an in-house perl script, and then quality-filtered by fastp version 0.19.6 and merged by FLASH version 1.2.7 with the following criteria: (i) The reads were truncated at any site receiving an average quality score of <20 over a 50 bp sliding window, and truncated reads shorter than 50 bp or reads containing ambiguous characters were discarded. (ii) Only overlapping sequences longer than 10 bp were assembled according to their overlapped sequence. The maximum mismatch ratio of overlap region was 0.2. Reads that could not be assembled were discarded. (iii) Samples were distinguished according to the barcode and primers, and the sequence direction was adjusted and exact barcode matching was performed, with a maximum 2-nucleotide mismatch in primer matching. Then, the optimized sequences were clustered into operational taxonomic units (OTUs) using UPARSE v7.0.1090 with 97% sequence similarity level. The most abundant sequence for each OTU was selected as a representative sequence. To minimize the effects of sequencing depth on alpha and beta diversity measurements, the number of 16S rRNA gene sequences from each sample was rarefied to 20,000, which still yielded an average Good’s coverage of 99.09%. The taxonomy of each OTU representative sequence was analyzed by RDP Classifier version 2.2 against the 16S rRNA gene database (e.g., Silva v138) using a confidence threshold of 0.7.

### 2.9. Statistical Analysis

Bioinformatic analysis of gut microbiota was carried out using the Majorbio Cloud platform (https://cloud.majorbio.com, accessed on 28 February 2025). Based on the OTUs information, rarefaction curves and alpha diversity indices including observed OTUs, Chao1 richness, Shannon index, and Good’s coverage were calculated using Mothur v1.30.1. The similarity among the microbial communities in different samples was determined by principal coordinate analysis (PCoA) based on Bray–Curtis dissimilarity using the Vegan v2.5-3 package. The PERMANOVA test was used to assess the percentage of variation explained by the treatment, along with its statistical significance, using Vegan v2.5-3 package. The linear discriminant analysis (LDA) effect size (LEfSe) (http://huttenhower.sph.harvard.edu/LEfSe, accessed on 28 February 2025) was performed to identify the significantly abundant taxa (phylum to genera) of bacteria among the different groups (LDA score > 4, *p* < 0.05).

IBM SPSS Statistics 22.0 software (version 22.0; IBM SPSS, Inc., Chicago, IL, USA) was used to conduct a one-way ANOVA test and one-dimensional variance descriptive statistical analysis on data such as body weight comparison and lesion scores from the six experimental groups of chickens, with (*p* < 0.05) as the significance judgment criterion.

## 3. Results

### 3.1. Growth Performance

**Figure 1:**
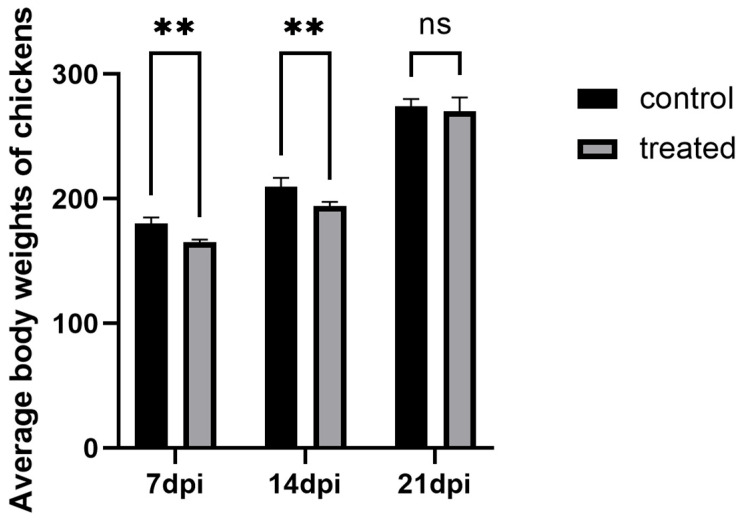
The comparison of average body weights of layer hens across experimental groups. ** *p* < 0.05, ns *p* > 0.05.

During the infection process, the chickens in each experimental group were weighed to obtain body weight comparison data. The results showed that significant differences in body weight were observed between the 7-day infected group and the control group, as well as between the 14-day infected group and the control group (*p* < 0.05). However, no significant difference was found in body weight in the 21-day experimental group (*p* > 0.05), suggesting that the severity of *H. meleagridis* infection was more pronounced at 7 days post-infection (dpi) and 14 days post-infection (dpi). The results are shown in Figure 1.

### 3.2. Intestinal Lesion and Intestinal Lesion Score

**Figure 2:**
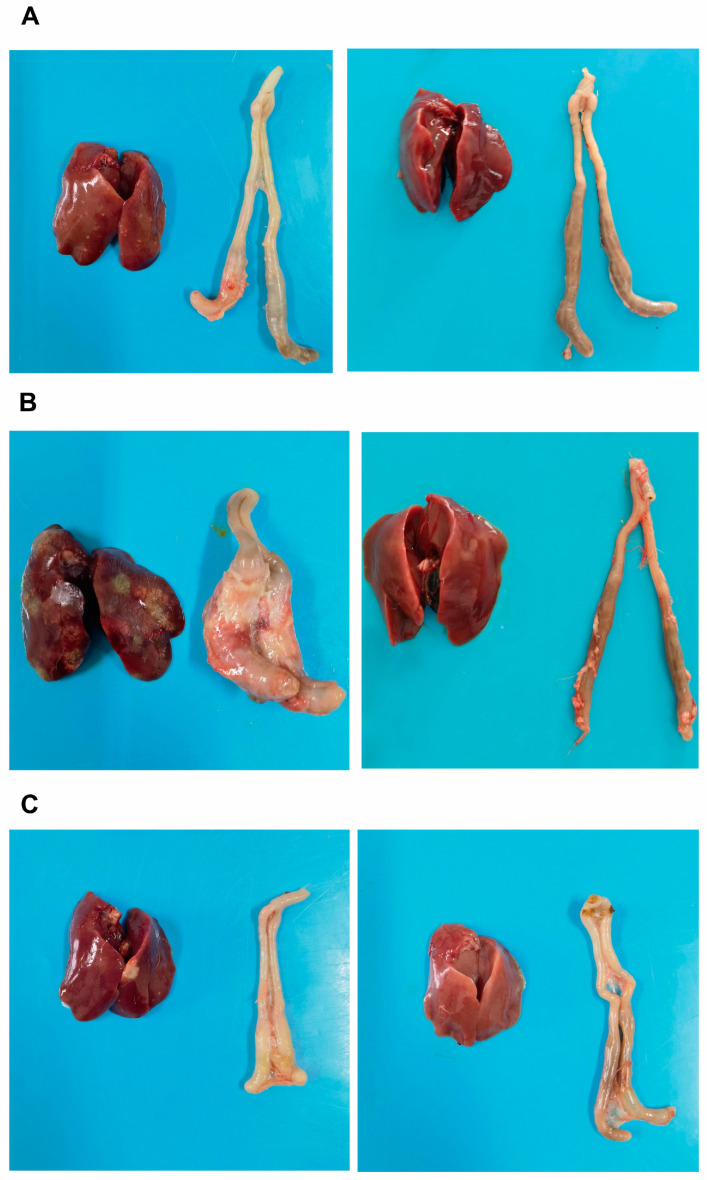
The comparison of liver and cecal lesion conditions across experimental groups. (**A**): Liver and cecum of the infected group (**left**) and control group (**right**) at 7 days post-infection (dpi); (**B**): Liver and cecum of the infected group (**left**) and control group (**right**) at 14 dpi; (**C**): Liver and cecum of the infected group (**left**) and control group (**right**) at 21 dpi.

The chickens in each experimental group were dissected to observe pathological changes. The results showed that, in the 7 dpi group, during necropsy, a small number of chickens had crater-like necrotic foci on the liver, with the diameter of the necrotic foci being less than 5 mm, and an obvious cecal plug (Figure 2A, the left side of the image represents the infected group, while the right side represents the control group; the same applies below). In the 14 dpi group, most chickens exhibited liver enlargement, obvious congestion, and multiple crater-like necrotic foci, with some necrotic foci exceeding 5 mm in diameter; the liver was brittle and fragile. The cecum was significantly swollen, with increased volume, and when cut open, the cecal wall was thickened, adhered, and a cecal plug was visible inside (Figure 2B). In the 21 dpi group, during necropsy, a small number of chickens had necrotic foci on the liver, and the cecum was slightly swollen (Figure 2C); no pathological changes were observed in the control group.

**Figure 3:**
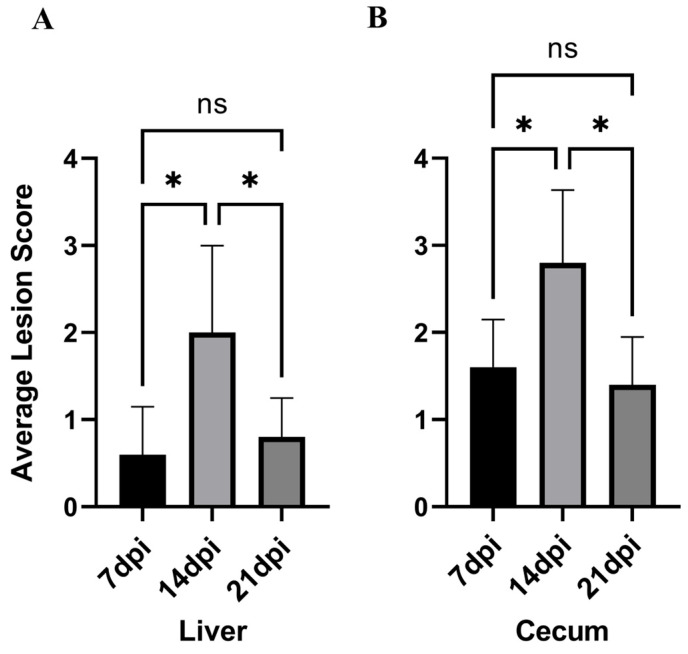
The lesion scoring status across experimental groups. (**A**): Liver lesion score. (**B**): Cecum lesion score. * *p* < 0.05, ns *p* > 0.05.

The average liver–cecum lesion scores of chickens in each experimental group are shown in Table 1 and Figure 3. It can be observed that the lesion score at 14 dpi is the highest, with the average liver–cecum lesion scores at 14 dpi significantly higher than those at 7 dpi (*p* < 0.05). Furthermore, the average liver–cecum lesion scores at 21 dpi were significantly lower than those at 14 dpi (*p* < 0.05).

Given that there was no significant difference in body weight between the infected and control groups at 21 days, and that the liver and cecal lesion scores were significantly reduced, we selected the following four groups for 16S rRNA sequencing analysis: the 7-day control group, the 7-day infected group, the 14-day control group, and the 14-day infected group.

### 3.3. Rarefaction Curve and the Change in the Alpha and Beta of Cecal Gut Microbiota

**Figure 4:**
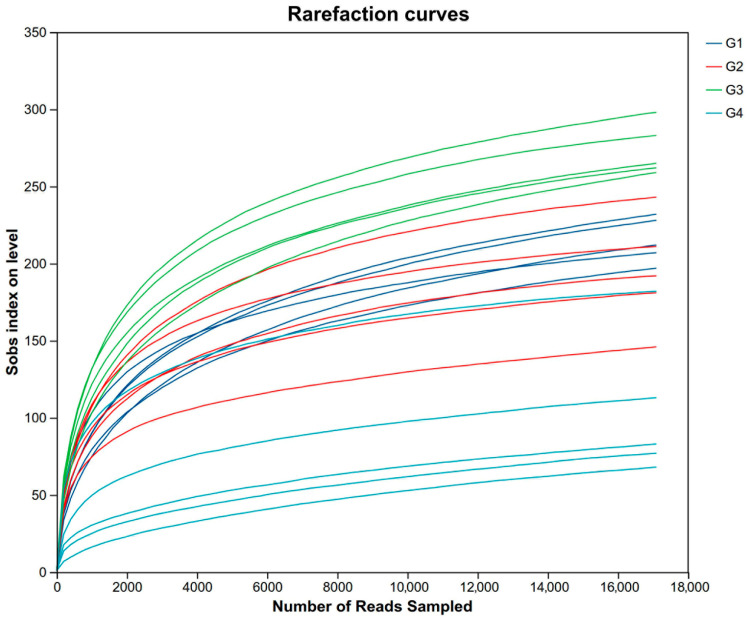
The rarefaction curves of 20 samples. Abbreviations: G1, the 7-day control group; G2, the 7-day infected group; G3, the 14-day control group; G4, the 14-day infected group.

For descriptive convenience, the 7-day control group, the 7-day infected group, the 14-day control group, and the 14-day infected group are, respectively, designated as G1, G2, G3, and G4. After quality filtering and assembly, we obtained a total of 669,538 sequences; the average sequence lengths were 1458 bp per sample. The rarefaction curves of 20 samples stabilized as the data increased, indicating sufficient data sampling and sequencing depth to assess richness and microbial community diversity (Figure 4).

**Figure 5:**
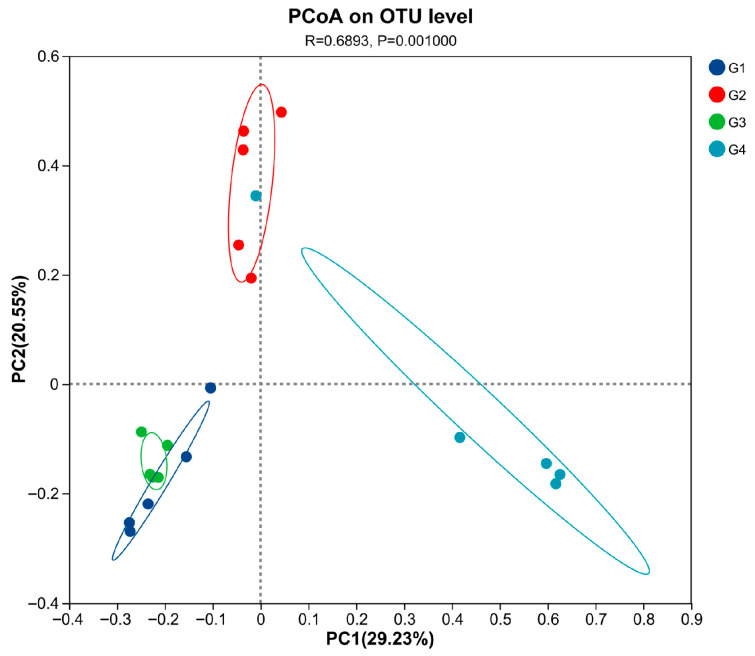
Principal coordinate analysis (PCoA) was performed at the OUT level based on weighted UniFrac distances for all samples. Abbreviations: G1, the 7-day control group; G2, the 7-day infected group; G3, the 14-day control group; G4, the 14-day infected group.

Compared with the control group (Table 2), there were no significant differences in the ACE, Chao, Shannon, and Simpson indices in the 7 dpi group (*p* > 0.05). The ACE, Chao, Shannon, and Sobs indices in the 14 dpi group were significantly decreased (*p* < 0.05). When comparing the 7 dpi group with the 14 dpi group, it was found that the ACE, Chao, Shannon, and Sobs indices at 14 dpi were significantly decreased (*p* < 0.05). The PCoA results (Figure 5) demonstrated significant differences in cecal microbiota diversity among the groups (*p* = 0.001). The cecal microbial structure distribution of the infected and control groups at 14 dpi showed distinct clustering on opposite sides, with significant separation. At 14 dpi, the microbial structure distribution of the cecal microbiota in the control and infected groups of chickens showed a clear left–right separation with a substantial distance between them. Compared to the control group, the infected group deviated toward the positive PC1 axis, and this deviation increased over time.

### 3.4. Analysis of the Composition and Differential Changes in Species Abundance of Cecal Microbiota in Chickens

**Figure 6:**
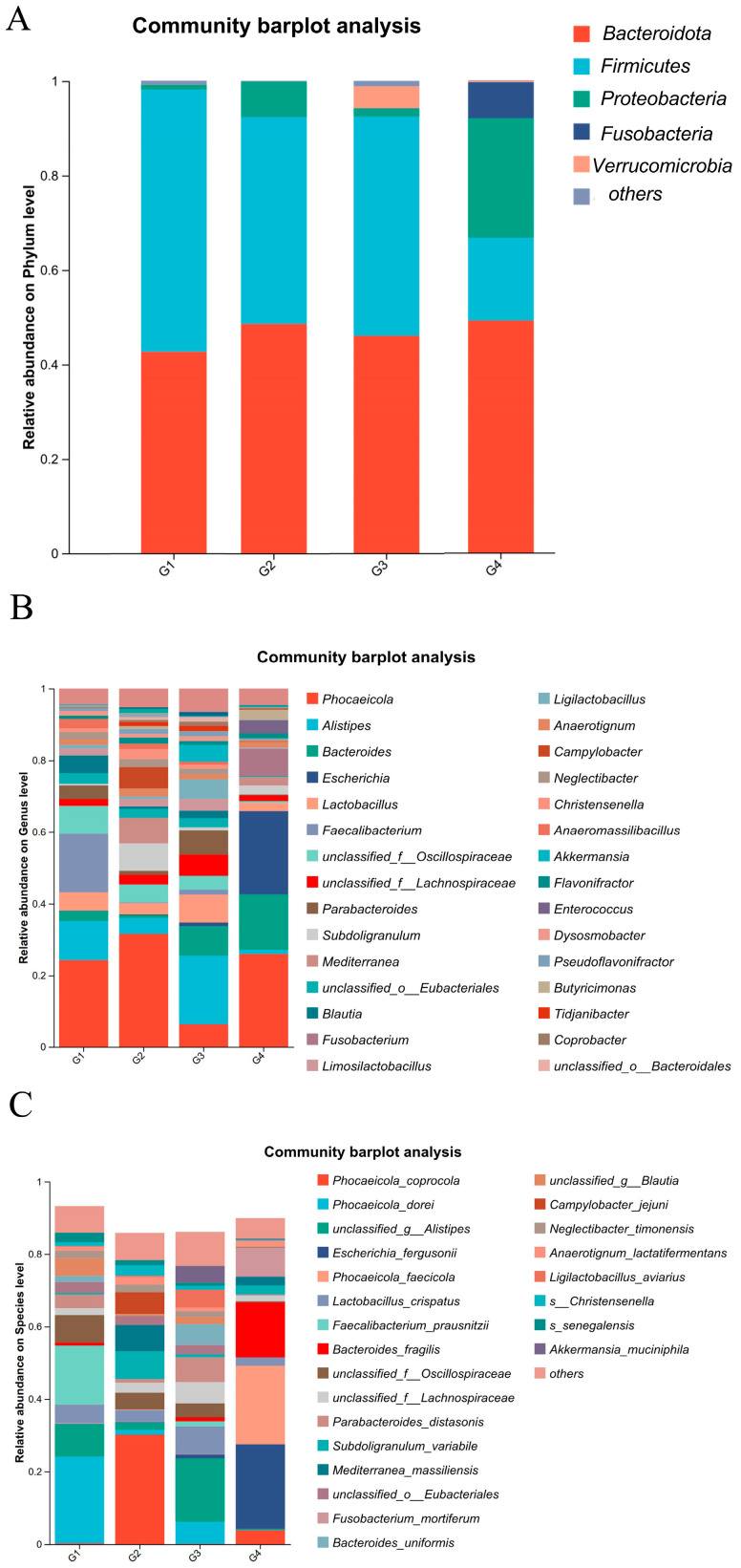
Composition of cecal microbiota species abundance. (**A**) The phylum level. (**B**) The genus level. (**C**) The species level. Abbreviations: G1, the 7-day control group; G2, the 7-day infected group; G3, the 14-day control group; G4, the 14-day infected group.

As illustrated in Figure 6A, at the phylum level, the dominant bacterial phyla in both the control and infected groups were *Bacteroidota*, *Proteobacteria*, and *Firmicutes*, with the additional presence of Fusobacteria and Verrucomicrobia. After *H. meleagridis* infection, the abundance of Firmicutes decreased, whereas the abundances of Bacteroidota and Proteobacteria increased. Verrucomicrobia was detected in the control group, while *Fusobacteria* emerged in the infected group. Compared to the 7 dpi group, the 14 dpi group showed a further reduction in Firmicutes abundance and an elevation in Proteobacteria abundance.

As shown in Figure 6B, at the genus level, the dominant bacterial genera in the chicken intestinal microbiota include *Phocaeicola* spp., *Alistipes* spp., *Bacteroides* spp., *Escherichia* spp., *Lactobacillus* spp., and *Faecalibacterium* spp. Following *H. meleagridis* infection, compared to the control group, the abundances of *Phocaeicola* spp., *Bacteroides* spp., *Escherichia* spp., *Subdoligranulum* spp., and *Fusobacterium* spp. increased, whereas the abundances of *Alistipes* spp., *Lactobacillus* spp., *Parabacteroides* spp., *Mediterranea* spp., *Blautia* spp., *Limosilactobacillus* spp., *Ligilactobacillus* spp., *Anaerotignum* spp., and *Campylobacter* spp. decreased. Compared to the 7 dpi group, the 14 dpi group exhibited increased abundances of *Bacteroides* spp., *Escherichia* spp., *Fusobacterium* spp., *Enterococcus* spp., and *Mediterranea* spp., while the abundances of *Alistipes* spp., *Lactobacillus* spp., *Akkermansia* spp., and *Limosilactobacillus* spp. decreased.

At the species level (Figure 6C), the dominant bacterial species in the chicken intestinal microbiota include *Bacteroides fragilis*, *Lactobacillus crispatus*, and *Faecalibacterium prausnitzii*. Following *H. meleagridis* infection, compared to the control group, the abundance of *Phocaeicola coprocola*, *Escherichia fergusonii*, *Bacteroides fragilis*, *Subdoligranulum variabile*, *Mediterranea massiliensis*, *Fusobacterium mortiferum*, *Anaerotignum lactatifermentans*, and *Campylobacter jejuni* increased, while the abundances of *Phocaeicola dorei*, *Phocaeicola faecicola*, *Lactobacillus crispatus*, *Faecalibacterium prausnitzii*, *Parabacteroides distasonis*, *Bacteroides uniformis*, *Ligilactobacillus aviravius*, and *Akkermansia muciniphila* decreased. Compared to the 7 dpi group, the 14 dpi group exhibited a marked increase in the abundance of *Escherichia fergusonii*, *Phocaeicola faecicola*, *Bacteroides fragilis*, *Mediterranea massiliensis*, and *Fusobacterium mortiferum*, whereas the abundances of *Phocaeicola coprocola*, *Lactobacillus crispatus*, *Subdoligranulum variabile*, and *Campylobacter jejuni* showed a pronounced decline.

### 3.5. LEfSe-Based Discriminant Analysis of Multi-Level Species Differences in Cecal Microbiota Across Experimental Groups

**Figure 7:**
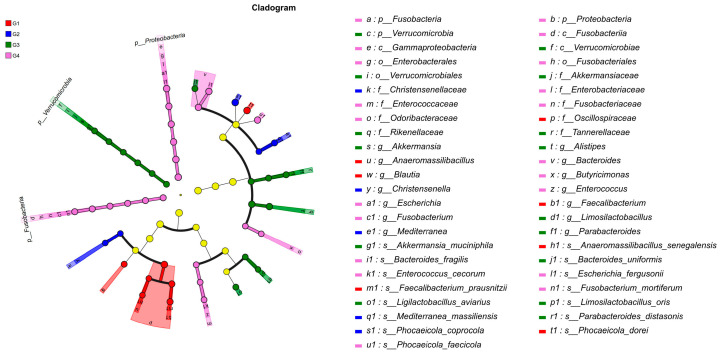
LEfSe analysis of taxonomic biomarkers in cecal microbiota among experimental groups. Abbreviations: G1, the 7-day control group; G2, the 7-day infected group; G3, the 14-day control group; G4, the 14-day infected group.

**Figure 8:**
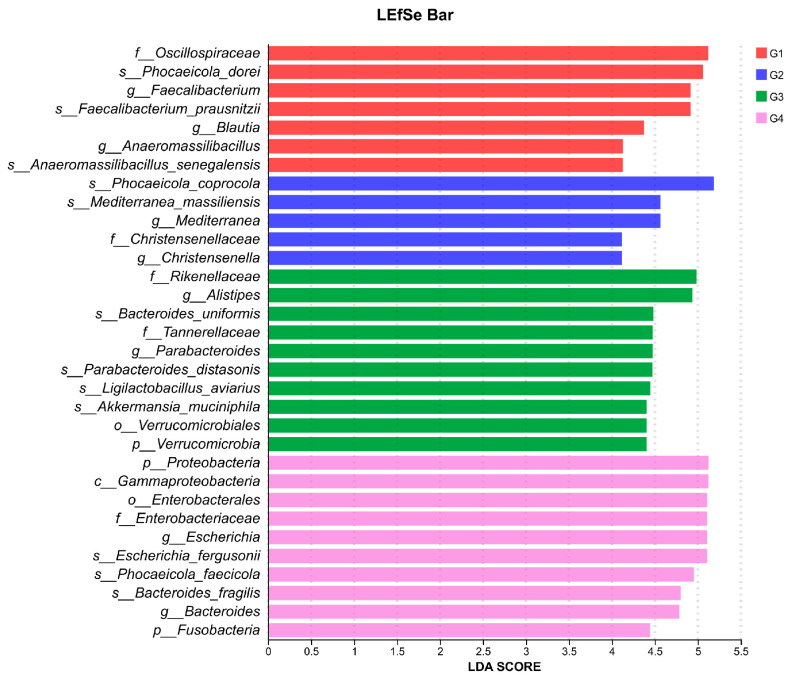
LDA scores obtained from the LEfSe analysis of the gut microbiota in different groups. An LDA effect size of greater than 4 was used as a threshold for the LEfSe analysis. Abbreviations: G1, the 7-day control group; G2, the 7-day infected group; G3, the 14-day control group; G4, the 14-day infected group.

To gain a comprehensive understanding of the impact of *H. meleagridis* infection on the gut microbiota, we conducted LEfSe analysis. The taxonomic cladogram and LDA scores obtained from the LEfSe analysis confirmed and visualized the effects of infection (Figure 7 and Figure 8). Filtered by LEfSe analysis (LDA threshold of 4), it was found that at 7 dpi, the control group was enriched with *Oscillospiraceae* spp., *Blautia* spp., *Phocaeicola dorei*, and *Faecalibacterium prausnitzii*, while the infected group was enriched with *Christensenella*, *Phocaeicola coprocola*, and *Mediterranea massiliensis*; at 14 dpi, the control group showed enrichment of Verrucomicrobia, *Rikenellaceae*, *Tannerellaceae*, *Akkermansiaceae*, *Alistipes* spp., *Bacteroides* spp., *Bacteroides uniformis*, *Ligilactobacillus aviarius*, and *Limosilactobacillus oris*, whereas the infected group exhibited enrichment of Proteobacteria, Fusobacteria, *Escherichia* spp., *Fusobacterium* spp., *Enterococcus* spp., *Escherichia fergusonii*, *Phocaeicola faecicola*, and *Bacteroides fragilis*.

## 4. Discussion

Histomonosis, a parasitic disease primarily affecting poultry, such as chickens and turkeys, is globally distributed and presents significant economic challenges to the poultry industry. The absence of prophylactic drugs and commercially available vaccines for treatment or prevention has led to increased efforts to control this disease [31]. Recent advancements in high-throughput sequencing technologies have enabled a more comprehensive understanding of the gut microbiota, which comprise trillions of microorganisms residing in the animal intestine [32]. A stable gut microbiota community is closely linked to host health, with the microbiota playing a pivotal role in modulating the immune system and enhancing production performance [18,33,34].

In this study, experimental infection of white leghorns with *H. meleagridis* revealed the most pronounced differences in body weight and lesion scores at 7 dpi and 14 dpi compared to controls, whereas lesions alleviated and body weight gradually recovered by 21 dpi, consistent with previous findings [35,36]. We hypothesized that these changes in body weight and pathology might be associated with alterations in the cecal microbiota induced by *H. meleagridis* infection. Therefore, cecal contents from infected chickens at 7 and 14 dpi were selected for microbiota diversity analysis. The observed weight recovery may be attributed to the resolution of infection and restoration of physiological functions. However, it should be noted that this study involved a limited sample size, which may introduce potential survivor bias or selection bias, warranting further investigation.

Analysis of microbiota diversity indicated that *H. meleagridis* infection induced marked changes in the cecal microbiota, which were likely associated with the severity of pathological lesions. We therefore hypothesize that reduced diversity of the cecal microbiota is accompanied by more severe cecal lesions and greater body weight loss, suggesting a potential negative correlation between cecal microbiota diversity and pathological damage. This indicates that dysbiosis of the cecal microbiota plays an important role in the pathophysiological changes induced by *H. meleagridis* infection.

Following *H. meleagridis* infection, the alpha diversity of the cecal microbiota in chickens decreased throughout the course of infection, indicating a reduction in both microbial richness and evenness. This phenomenon is consistent with findings from studies on multiple intestinal parasites. Infection with *E. tenella* and *E. necatrix* have been shown to reduce the diversity of the cecal microbiota in chickens [37,38]. Similarly, *Cryptosporidium* infection causing diarrhea in goats and calves led to decreased gut microbiota diversity [39,40]. To elucidate the dynamic distribution of bacterial species, this study further analyzed bacterial composition at the phylum, genus, and species levels. The most frequently identified bacteria in the cecal microbiota of control group chickens were Firmicutes, Bacteroidetes, Proteobacteria, Verrucomicrobia, and Fusobacteria, consistent with previous reports by Huang et al. [41]. This study revealed that following *H. meleagridis* infection, the abundances of Firmicutes and Verrucomicrobia decreased, while those of Proteobacteria, Bacteroidetes, and Fusobacteria increased. A higher ratio of Firmicutes to Bacteroidota helps reduce pathogen colonization [42]. In this case, the ratio decreased, while the levels of inflammatory bacteria, such as Proteobacteria and Fusobacteria, increased [43,44,45]. Based on the aforementioned changes, we propose that *H. meleagridis* infection alters the abundance of beneficial and pathogenic bacteria in the gut, which may influence the progression of histomonosis.

Following *H. meleagridis* infection, alterations were observed in the cecal microbiota at both the genus and species levels. Based on the cecal species composition, the abundances of *L. crispatus*, *L. aviarius*, *Limosilactobacillus vaginalis*, *L. oris*, and *Limosilactobacillus reuteri* were observed to decrease post-infection. Certain species of *Lactobacillus* spp. are well-established probiotics widely used in food and feed additives for disease prevention and treatment [46,47]. They ferment carbohydrates to produce lactic acid and short-chain fatty acids (SCFAs), secrete antimicrobial factors [48], promote mucus and sIgA secretion [49], modulate immune cytokine expression [50,51,52], and alter gut microbiota composition, thereby exerting antibacterial, anti-inflammatory, and intestinal barrier-enhancing effects [53]. *L. crispatus* belongs to the genus *Lactobacillus*, is commonly found in the female urogenital tract, and helps combat colonization by uropathogens [54]. In livestock and poultry, *L. crispatus* produces lactic acid and lowers intestinal pH to inhibit the growth of harmful bacteria such as *C. jejuni* and *Salmonella* [55,56]. Additionally, *L. aviarius*, *L. vaginalis*, *L. oris*, and *L. reuteri* belong to the genera *Ligilactobacillus* spp. and *Limosilactobacillus* spp. within the phylum *Firmicutes*. The combined application of *L. salivarius* and *L. reuteri* in broilers has been shown to enhance intestinal morphology and increase the expression of the anti-inflammatory cytokine IL-10 [57]. Furthermore, *L. reuteri* and *L. vaginalis* isolated from chicken ceca produce extracellular polysaccharides (EPSs), which facilitate intestinal colonization of beneficial bacteria and exhibit strong antibacterial activity against *E. coli* and *Salmonella Typhimurium* in vitro [58]. Based on these findings, we hypothesize that *H. meleagridis* infection may lead to a relative reduction in the abundance of these species, thereby decreasing the secretion of anti-inflammatory factors and lactic acid, weakening the intestinal barrier. This could create opportunities for the colonization of opportunistic pathogens, consequently exacerbating pathological lesions.

In addition to the genus *Lactobacillus*, beneficial bacterial species such as *Alistipes*, *F. prausnitzii*, and *A. muciniphila* were also notably present in the control group. Similarly to *Lactobacilli*, *Alistipes* is a propionate producer capable of expressing acetyl-CoA carboxylase. It also expresses glutamate decarboxylase, influencing SCFA levels and the production of the metabolite γ-aminobutyric acid (GABA) [59]. *F. prausnitzii* produces butyrate with anti-inflammatory properties, increases the secretion of the anti-inflammatory cytokine IL-10 to reduce intestinal inflammation, and exerts anti-inflammatory effects by inhibiting the Th17 pathway [60,61]. *A. muciniphila*, belonging to the phylum *Verrucomicrobia*, degrades mucin, enhances SCFA levels, and contributes to intestinal barrier protection and immune regulation [62]. A significant reduction in the abundance of these bacterial species was observed at 14 dpi. We hypothesize that the intestinal inflammation caused by *H. meleagridis* infection may be related to the decreased abundance of beneficial bacteria possessing anti-inflammatory functions.

In addition to the beneficial bacteria mentioned above, changes in certain pathogenic bacteria are also noteworthy. As the infection progressed, the abundance of *Campylobacter* increased at 7 dpi. *C. jejuni*, the predominant species within this genus and a zoonotic pathogen, employs capsular polysaccharide (CPS) production to evade the host immune system [63]. However, existing studies have demonstrated that lactobacilli possess anti-Campylobacter properties and can reduce *C. jejuni* loads [62]. By 14 dpi, the abundances of *Escherichia* spp. and *Fusobacterium* spp. increased, specifically manifested as a rise in *E. fergusonii* and *F. mortiferum*. Certain species of *Fusobacterium* can invade colonic epithelial cells and activate intracellular inflammatory signaling pathways [64]. A research indicated that *E. fergusonii* carries genes encoding the virulence factor heat-labile enterotoxin (LT), which is also present in enterotoxigenic *E. coli* (ETEC) [65]. This toxin penetrates cells and induces apoptosis in intestinal epithelial cells, although its activity can be suppressed by SCFAs [66]. In this study, we observed that *C. jejuni* increased significantly at 7 dpi but markedly decreased at 14 dpi, which we hypothesize may be suppressed by *lactobacilli*—particularly *L. crispatus*—consistent with earlier findings. Conversely, the notable increase in *E. fergusonii* and *F. mortiferum* at 14 dpi may have resulted from the reduction in beneficial bacterial populations. Therefore, we propose that the decline of these beneficial species likely impaired microbial metabolite levels and cytokine-mediated immune pathways, thereby leading to compromised intestinal barrier function and exacerbated inflammatory responses. As a consequence, more severe pathological manifestations were observed during the later stages of *H. meleagridis* infection. Although certain functional correlations of the aforementioned bacterial species have been described, this study lacks supporting functional data, such as metagenomics analyses, SCFA quantification, and transcriptomic profiling. Hence, the observed associations between these bacteria and histomonosis should be regarded as preliminary inferences based on current observations, and their functional relevance warrants further investigation.

Through LEfSe analysis, we observed that at 7 dpi, the control group was predominantly enriched with bacterial taxa possessing potential probiotic functions, including key butyrate- and acetate-producing genera such as *Blautia* and *F. prausnitzii*. The latter is recognized as a crucial marker of intestinal health, renowned for its anti-inflammatory properties, such as inducing regulatory T cells and generating anti-inflammatory metabolites [67]. Additionally, *P. dorei* and *L. reuteri* have also been associated with the maintenance of gut homeostasis [68,69]. In contrast, the 7 dpi group showed enrichment of *Christensenellaceae*. While *Christensenellaceae* is often linked to host leanness and is known to influence gut microbiota composition [70], with some studies indicate that its abundance may decrease under disease conditions [71]. Therefore, we hypothesize that early-stage *H. meleagridis* infection impairs the enrichment of beneficial bacteria in the gut, which may lead to impaired intestinal health and body weight loss in chickens.

As the infection progressed to 14 days, the control group exhibited enrichment in various taxa associated with mucosal health and complex carbohydrate metabolism. These included *Akkermansiaceae* (particularly *A. muciniphila*), a representative of the Verrucomicrobia phylum, which plays a key role in maintaining intestinal barrier function by degrading and promoting regeneration of the mucus layer [72,73], as well as members of the *Bacteroides* spp., which help prevent intestinal inflammation through production of IL-10 and acetate [74]. Conversely, the 14 dpi group demonstrated a marked increase in pathogenic bacteria. Enrichment of Proteobacteria is a classic signature of gut dysbiosis [75]. Among its members, the genus *Escherichia* (including the species *E. fergusonii*) represents an emerging zoonotic pathogen that has been isolated from chicken meat in certain regions and from human patients with diarrhea. This bacterium can express the heat-labile enterotoxin (LT) gene [76,77] and participates in carbon source metabolism, playing a significant role in host energy accumulation [78,79]. More notably, the enrichment of Fusobacteria and its representative *Fusobacterium* spp. is particularly significant, as this phylum has been associated with colorectal cancer in humans [80] and can elevate levels of cytotoxic (CD8^+^) T cells to enhance anti-tumor responses. Additionally, the enrichment of *Bacteroides fragilis* warrants attention, as certain subtypes (such as enterotoxigenic ETBF) are established pathogenic factors [81]. Based on the above research findings, we hypothesize that under *H. meleagridis* infection, the abundance of pathogenic bacteria in the chicken gut increases, while the populations of metabolic and beneficial bacterial species decrease. This may adversely affect the growth performance and intestinal barrier function of chickens, leading to aggravated pathological severity.

The LEfSe analysis clearly delineates the structural changes in the gut microbiota induced by *H. meleagridis* infection: the early stage is characterized by a relative reduction in commensal/beneficial bacteria and an increase in specific bacterial families, while the later stage transitions to a dysbiotic state marked by a significant increase in Proteobacteria (particularly *Escherichia*), Fusobacteria, and opportunistic pathogens such as *B. fragilis*. We propose that this microbial shift may be linked to the pathological progression of *H. meleagridis* infection and the exacerbation of intestinal inflammation. However, our studies have indicated that LEfSe analysis requires a substantial volume of sequencing data to yield accurate results [82,83]. In contrast, the data sample size utilized in this study is relatively limited, which imposes constraints on identifying biomarker species affected by *H. meleagridis*. Therefore, further investigation is warranted.

## 5. Conclusions

In summary, this study demonstrates that *Histomonas meleagridis* infection results in reduced body weight, as well as cecal and hepatic lesions in chickens, leading to decreased species richness in the cecal microbiota, a reduced alpha diversity index of the gut microbial community, and alterations in the composition and structure of the cecal microbiome. Notably, the abundance of non-pathogenic bacteria significantly diminished. Conversely, the abundance of opportunistic pathogens increased. This research provides a more comprehensive theoretical foundation for understanding the interactions between parasites and gut microbiota. Furthermore, these findings may offer new strategic insights for preventing and controlling histomoniasis through microbial intervention strategies. However, the findings of this study still require validation with larger sample sizes and more functional data.

## Figures and Tables

**Table 1:** The lesion scoring status across experimental group.

Types	7 dpi	14 dpi	21 dpi
Liver	0.60 ± 0.54 ^a^	2.00 ± 1.00 ^b^	0.80 ± 0.45 ^a^
Cecum	1.60 ± 0.55 ^a^	2.80 ± 0.84 ^b^	1.40 ± 0.24 ^a^

Data with different letters indicates significant difference (*p* < 0.05).

**Table 2:** Comparative analysis of alpha diversity indices across experimental groups.

Group	ACE	Chao1	Shannon	Simpson	Coverage	Sobs
G1	255.8 ± 23.23 ^a^	263.5 ± 25.10 ^a^	2.927 ± 0.412 ^a^	0.1431 ± 0.0563 ^a^	0.9972 ± 0.00052 ^a^	215.2 ± 14.62 ^a^
G2	233.1 ± 28.11 ^a^	226.4 ± 28.36 ^a^	2.954 ± 0.410 ^a^	0.1905 ± 0.0863 ^a^	0.9972 ± 0.00052 ^a^	194.6 ± 35.93 ^a^
G3	310.9 ± 14.98 ^b^	312.6 ± 18.42 ^ab^	3.561 ± 0.160 ^a^	0.0744 ± 0.0075 ^a^	0.9971 ± 0.00037 ^a^	273.4 ± 16.62 ^b^
G4	155.7 ± 28.66 ^c^	135.4 ± 35.78 ^c^	2.059 ± 1.059 ^ab^	0.2875 ± 0.2749 ^a^	0.9983 ± 0.00018 ^ab^	104.6 ± 46.45 ^c^

Data with different letters indicates significant difference (*p* < 0.05). Abbreviations: G1, the 7-day control group; G2, the 7-day infected group; G3, the 14-day control group; G4, the 14-day infected group.

## Data Availability

The original data presented in the study are openly available. Raw sequence reads were uploaded to the NCBI BioProject databank (PRJNA 1338818).
